# Non-invasive Respiratory Support of the Premature Neonate: From Physics to Bench to Practice

**DOI:** 10.3389/fped.2020.00214

**Published:** 2020-05-08

**Authors:** Ibrahim Sammour, Sreenivas Karnati

**Affiliations:** Department of Neonatology, Lerner College of Medicine, Pediatric Institute, Cleveland Clinic, Cleveland Clinic Foundation, Cleveland, OH, United States

**Keywords:** non-invasive ventilation, CPAP, bubble CPAP, nasal high frequency ventilation, high flow nasal cannula, neurally adjusted ventilator assist (NAVA), Bronchopulmonary dysplasia

## Abstract

Premature births continue to rise globally with a corresponding increase in various morbidities among this population. Rates of respiratory distress syndrome and the consequent development of Bronchopulmonary Dysplasia (BPD) are highest among the extremely preterm infants. The majority of extremely low birth weight premature neonates need some form of respiratory support during their early days of life. Invasive modes of respiratory assistance have been popular amongst care providers for many years. However, the practice of prolonged invasive mechanical ventilation is associated with an increased likelihood of developing BPD along with other comorbidities. Due to the improved understanding of the pathophysiology of BPD, and technological advances, non-invasive respiratory support is gaining popularity; whether as an initial mode of support, or for post-extubation of extremely preterm infants with respiratory insufficiency. Due to availability of a wide range of modalities, wide variations in practice exist among care providers. This review article aims to address the physical and biological basis for providing non-invasive respiratory support, the current clinical evidence, and the most recent developments in this field of Neonatology.

## Introduction

The importance of respiratory failure as a cause of mortality in newborns has been recognized as early as 2750 BC by Huang Ti; a Chinese philosopher and emperor. The use of positive end-expiratory pressure (PEEP) and continuous positive airway pressure (CPAP) to support premature infants heralds back to the early 1900s with Hoerder recommending a tracheal intubation with what amounts to a bubble CPAP system to generate positive end-expiratory pressure, and Engelmann describing the use of facial CPAP to support a distressed infant. These early interventions were recorded in the textbook Diseases of the Newborn written by Dr. August Ritter von Reuss, with the original German text being published in 1914, and the English translation being released in 1922 (1).

Over the past 2 decades the incidence of premature births continued to increase worldwide, with the most recent estimate pointing to around 15 million premature births around the globe annually. This increase was accompanied by improved survival of the most premature of infants. Despite these improvements, preterm birth was the leading cause of neonatal deaths, and second in line for early childhood deaths in the first 5 years of life, superseded only by infectious agents (2, 3). Preterm birth is also associated with a number of morbidities that affect the life expectancy and quality of life of these children. One of the more serious morbidities is chronic lung disease of prematurity (CLD) or Bronchopulmonary dysplasia (BPD). Rates of BPD increase with decreasing gestational age, with half of the children born at <26 weeks' gestation afflicted (4). Developing BPD is associated with long term respiratory morbidities and pulmonary arterial hypertension (5–8).

Newborns require respiratory support for several reasons including pulmonary insufficiency of prematurity, apnea of prematurity, respiratory distress syndrome, disorders of transitioning, and persistent pulmonary hypertension to name a few. Non-invasive respiratory support has been used after extubation to reduce extubation failures, or as a primary modality for infants deemed to be stable enough. Modalities utilized in a non-invasive manner to support neonates include various CPAP devices, non-invasive positive pressure ventilation / non-invasive mandatory ventilation (NIPPV and NIMV, respectively), high flow nasal cannula (HFNC), and more recently high frequency nasal ventilation (HFNV).

The pathophysiology of BPD is complicated and multifactorial. One of the more pronounced risk factors is gestational age at birth, with infants born earlier during the canalicular phase being at highest risk for injury. Other risk factors identified over the past 2 to 3 decades include various genetic factors, infections, systemic inflammatory conditions, and prolonged invasive ventilatory support have all been implicated in the development of BPD. Given the profound effect of invasive mechanical ventilation, different invasive and non-invasive ventilatory strategies have been explored in an attempt to reduce lung injury and BPD.

## Basics of Gas Physics and Ventilation

No complete discussion of non-invasive support can be made without touching upon the physics that govern ventilation. During laminar flow, the resistance to flow through a tube is governed by Poiseuille's law denoted below.

$$\begin{array}{l} R=\frac{8\eta L}{\pi r^{4}} \end{array}$$

Where R is resistance, η is viscosity, L is tube length, and r is tube radius. As the length of a tube increases, resistance increases in a linear manner. Moreover, a reduction in radius will cause an exponential increase in resistance. Conversely the laminar flow through a tube is denoted by the equation below

$$\begin{array}{l} \overset{∙}{V}=\frac{P1-P2}{R}=\frac{\pi \left(P1-P2\right)r^{4}}{8\eta L} \end{array}$$

In the equation above, V° denotes flow, P1-P2 denotes a pressure difference, with the rest of the symbols being listed above. For flow to occur, a pressure gradient needs to exist, and that the amount of flow generated by a pressure differential is inversely related to the resistance of that system.

Furthermore, the pressure built up within a system is covered by the equation of motion below.

$$\begin{array}{l} Pressure=Elastance.Volume+Flow.Resistance \end{array}$$

The pressure within a respiratory system; the patient and the supporting circuit, is determined by the elastance of that system as a whole, the volume of gas injected into it, the flow through it, and the resistance of said system to flow. A system that is less compliant, with a higher elastance, will build up a greater amount of pressure for a given volume of gas injected. Whereas, a system posing a higher resistance due to either longer or small tubing or airways, will build more pressure for a given flow. Conversely if flow through a system is increased, the pressure will rise.

**Figure 1:**
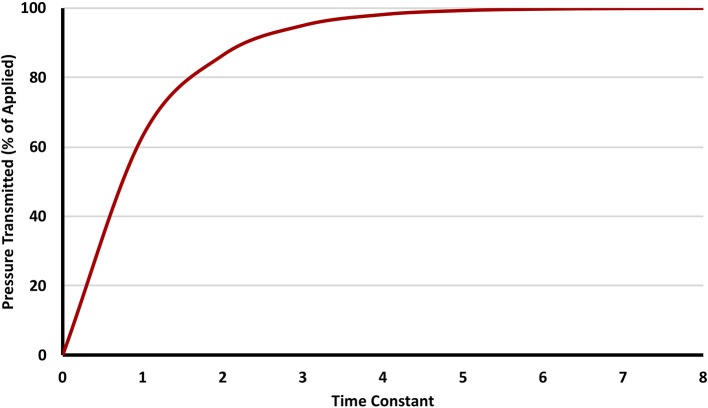
The relationship between time constant and delivered pressure. Three to five time constants are needed to transmit >95% of the pressure applied.

Assuming no leak within a system, when gas flows from a point of higher pressure to another of lower pressure, the pressure on the receiving end increases over time, and eventually stops once pressure equilibrates. The time necessary for equilibration is governed by the time constant (TC). The value of the TC in seconds is the product of multiplying compliance by resistance. Its value denotes the time needed for 63% of the pressure gradient to transmit from one point to another (Figure 1 shows the transmission of an applied pressure as multiple time constants pass). A system with a significant leak can be thought of as having near-infinite compliance, and hence cannot be pressurized easily.

$$\begin{array}{l} Time\;Constant=Compliance.Resistance \end{array}$$

## Components of a Non-Invasive Respiratory Support, Their Impact, and Technical Aspects

Different components are needed to provide respiratory support; a source of pressurized gas, a device to regulate either flow or pressure, a humidifier, a breathing circuit, and a nasal interface. Devices used to provide continuous distending pressure can be divided into variable or constant flow drivers. A variable flow driver adjusts flow to achieve pressure in the face of reasonable leak; examples of such devices include various ventilators, and devices like the Infant Flow nCPAP system (Vyaire Medical, CareFusion, Illinois, USA). Constant flow drivers utilize a resistor to build up pressure. These resistors can be of a constant value as observed in SiPAP machines or “threshold” in nature and hence prevent pressure from building up beyond the set pressure as seen in bubble CPAP (bCPAP) devices. This threshold behavior of bCPAP devices generates relatively random high frequency oscillations of the order of 15 to 30 Hz with varying amplitudes depending on flow used (9).

### CPAP Generators

The use of CPAP in neonates can be traced back to 1970s when different devices and interfaces were developed for use in neonates (10). Since that time, these devices were revised and iterated upon numerous times, and CPAP is now recommended by the World Health Organization (WHO) as a first-line therapy to support premature neonates worldwide (11).

**Figure 2:**
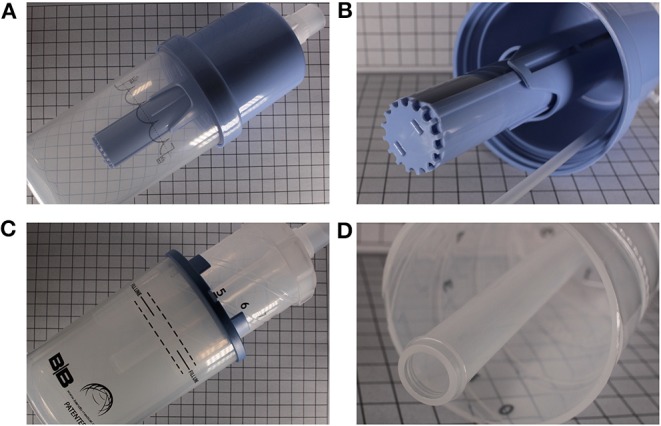
Two different commercially available bubblers. **(A)** Babi.Plus Bubble PAP Valve (GaleMed), with the diffuser at the expiratory end **(B)**. **(C)** B and B Bubbler (B and B Medical Technologies) without a diffuser at the expiratory end **(D)**.

In a benchtop study of 3 contemporary bCPAP systems and a homemade one, Poli et al. demonstrated that differently constructed bubblers generated different pressure amplitudes, with these amplitudes being flow-dependent in all but one system. Furthermore, they demonstrated that the addition of a diffuser to the opening of the expiratory tube blunted the amplitude of these oscillations (Figure 2 demonstrates the appearance of CPAP devices with and without diffusers in place) (12). These design variations can potentially affect the efficacy of a bCPAP system in clearing CO_2_.

The importance of the signal noise in a bCPAP was further illustrated by Sivieri et al. using a benchtop premature infant lung model. They studied the effect of superimposing an in-line high frequency signal using a flow interrupter to both ventilator derived CPAP (nCPAP) and bCPAP. The addition of high frequency oscillations to nCPAP and its superimposition onto bCPAP Increased CO_2_ removal as evidenced by a decline in end-tidal CO_2_ (13).

### Devices for NIMV

The application of intermittently higher pressure over positive end-expiratory pressure (PEEP) is known by many terms including Bi-Phasic, BiPAP, Non-invasive positive pressure ventilation (NIPPV), non-invasive mechanical ventilation and non-invasive mandatory ventilation (NIMV). Despite the differences in nomenclature and devices used to deliver these pressures, this doesn't affect the underlying physics.

When considering the use of a ventilator or dedicated NIMV device to provide non-invasive respiratory support, one must first take into account its leak compensation (LC) capabilities. LC is the ability to adjust the flow to maintain the pressure within the circuit in the face of significant leak. The importance of LC algorithms and their ability to impact synchrony during NIMV was highlighted by Itagaki et al. through a benchtop lung model (14). Using a lung simulator (ASL 5000 from IngMar Medical, Pittsburgh, Pennsylvania) the team demonstrated that in non-invasive modes, most of the ventilators tested failed to synchronize when a leak was present. Their study was limited however by the use of the Neotech RAM cannula which imposes a higher resistance to flow than interfaces designed for pressure transmission (15, 16). Other considerations should include the performance characteristics of exhalation valves. Exhalation valves can be passive or active. An active exhalation valve allows a patient to exhale back into the circuit during an IMV breath without causing the pressure to rise up beyond the set peak inflating pressure. Not all ventilators possess active exhalation valves, and not all of them behave in a similar manner as demonstrated by Jiao et al. (17).

A special form of NIMV known as Neurally Adjusted Ventilator Assist (NAVA) has started to gain traction in NICUs. This proprietary mode only available on the Servo-I and Servo-U ventilators (Getinge, NJ, USA), utilizes a specialized orogastric tube lined with sensors which are used to detect the electrical activity of the diaphragm (EAdi). This signal is generated as the myoelectric impulse propagates throughout the diaphragm, before any detectable contraction and flow occurs. The ventilator then provides a proportional pressure assist to the patient's electromuscular signal using a simple multiplier referred to as the NAVA level. The use of EAdi rather than pneumatic sensors reduces asynchrony, peak inspiratory pressures and FiO_2_ requirements in invasively supported infants (18). Furthermore, should a signal not be detected due to apnea or a displaced catheter, the ventilator will fall onto a “backup” NIMV mode after a set “apnea time” passes. These features have sparked interest in its utility for non-invasive respiratory support.

Interestingly, rudimentary ventilators that use a bubble system with two different levels have been explored as means of potentially providing invasive and non-invasive respiratory support. Such devices would combine a bi-level mode of respiratory support with the noisy signal generated by bubbling through the circuit, however, no clinical studies have been conducted to explore their clinical efficacy (19, 20).

### Heated-Humidified High Flow Nasal Cannulas

Heated-humidified High flow nasal cannulas (HFNC) have been purported to provide respiratory support by flushing dead space continuously as demonstrated using a benchtop resin model of a nasal cavity by Spence et al. Using stereoscopic particle image velocitometry the team was able to demonstrate that HFNC was capable of continuously flushing the nasopharyngeal dead space, which theoretically would reduce the amount of carbon dioxide breathed back, and increased the rebreathed oxygen fraction (21).

Whether continuous flow HFNC is most beneficial in CO_2_ washout has recently been challenged by a benchtop study by Sivieri et al. Using a 3-way solenoid valve, they constructed a high frequency flow-interrupted HFNC. Testing frequencies between 4 and 10 Hz, they demonstrated a greater reduction in end-tidal CO_2_ during oscillatory HFNC when compared to continuous HFNC (22).

In a HFNC circuit, the pressure built up within is influenced by the set flow, resistance, leak around the nares and through the mouth, and the presence or absence of pressure-relief valves. Given that most HFNC systems are attached to high pressure hospital outlets, should a relief valve not be used or be defective, these pressures can potentially be transmitted to infants if an adequate leak is not present (23–25).

### The Breathing Circuit

An often-neglected component when discussing respiratory support is the actual respiratory circuit. Many infant respiratory circuits in use have a heated and humidified inspiratory limb, and a non-heated expiratory limb. Condensation can build up in the expiratory limb as well-humidified air cools down. Youngquist et al. demonstrated that the sloshing movement of condensed water in the expiratory limb could inadvertently increase the mean tracheal pressure, and oscillation amplitudes. The addition of a pressure-relief valve into the circuit was able to ameliorate these changes (26). Their findings support closer monitoring of respiratory circuits for condensation with regular evacuation of built up water.

### The Non-invasive Interfaces

**Figure 3:**
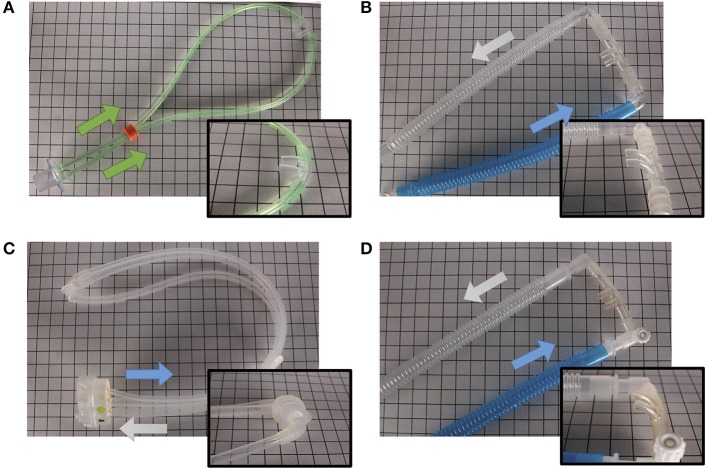
Different nasal devices. **(A)** RAM Cannula (NeoTech Products LLC), **(B)** Babi.Plus nCPAP Nasal Prongs (GaleMed), **(C)** NeoPAP Nasal Mask (Circadiance Pediatric Care), **(D)** Hudson Nasal Prongs (Teleflex). Arrows denote direction of air flow.

**Figure 4:**
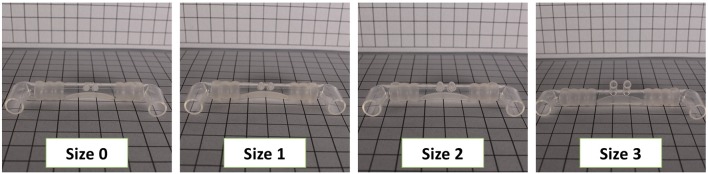
Different nasal prong sizes are needed to achieve a seal. Shown are Sizes 0 through 3 of the Babi.Plus nCPAP system.

Various interfaces are available to provide non-invasive support. Some interfaces can be attached to standard infant ventilator circuits such as the Hudson and INCA nasal prongs, others require their own unique circuit and delivery device such as the Infant Flow SiPAP system, which has interchangeable nasal masks and prongs. The design of the interface dictates its pressure transmission capabilities, ease of application, and potential for nasal injury. To transmit pressure adequately, interfaces need to form a good seal (Figures 3, 4 show some of the clinically available interfaces in North America). Furthermore, when a noisy signal is being transmitted through the interface; either in bCPAP or HFNV, it is important to recognize that it may be dampened by the design (27).

It is important to consider the differences in “pulmonary” mechanics when a nasal or facial interface is used instead of an endotracheal tube. In a study by Van Vonderen et al. they demonstrated a significant increase in inhaled and exhaled tidal volumes when a facial mask is used instead of an endotracheal tube in lambs and preterm infants. This was accompanied by an increase in leak, and no significant change in pressure transmitted (28).

Without a good seal, interfaces will deliver inconsistent pressures. By design, HFNC interfaces are non-occlusive to allow the entrainment of air around the cannula to accommodate varying flow demands during the respiratory cycle. When a high flow nasal cannula device is used contrary to its FDA approved indication, pressure is unlikely to be adequately transmitted. This was demonstrated by multiple studies where the NeoTech RAM cannula failed to deliver set CPAP and NIMV pressures (16, 29–31). The effect was further elucidated clinically by Matlock et al. where asynchronous NIMV did not yield any significant tidal volume delivery in 15 infants when their breaths did not coincide with machine breaths (32).

An ideal nasal interface would be easy to apply, maintain on the infant's face, of low resistance and low compliance, achieve a perfect seal without any leak, and would not result in nasal injury with prolonged use. None of the devices on the market as of this writing fulfill the criteria of an ideal nasal interface.

## Biological Effects of Distending Pressure in Animal Models

The disparities between devices used to provide non-invasive support and their clinical use render generalizing their protective effects difficult, however this is contrasted by the consistent injury demonstrated by invasive conventional mechanical ventilation in animal studies.

### CPAP and bCPAP

To study the immediate effects of the application of distending pressure compared to invasive mechanical ventilation Jobe et al. exposed preterm lambs to 2-h of invasive bCPAP or mechanical ventilation. The application of bCPAP was associated with a decrease in neutrophil migration into and H_2_O_2_ production in bronchoalveolar lavage (33).

Prolonged use of nCPAP in premature baboons (128/156 days) for 28 days, resulted in similar pulmonary mechanics, alveolarization, and close to normal vascular development when compared to term-borns. These findings suggest that arrest in alveolar development can be potentially prevented by using nCPAP (34). Subsequently, Thomson et al. went on to demonstrate that delaying extubation by as little as 5 days to CPAP was associated with worsened oxygenation and CO_2_ clearance when compared to animals extubated earlier. On the other hand, earlier extubation to nCPAP was associated with improved alveolarization, and decreased expression of pro-inflammatory mediators (35).

The importance of sustained lung expansion had been further elucidated in a study by Zhang et al. In juvenile ferrets, the sustained application of bCPAP for 2 weeks increased total lung capacity by 40%, with a significant increase in lung weight, total protein and DNA content, suggesting a promotion in lung growth (9).

The physiologic impact of the noisy signal generated by bCPAP systems was explored by Pillow et al. Utilizing a preterm lamb model of BPD and invasive application of bCPAP or nCPAP, they demonstrated the ability of bCPAP to better support the ventilation of these animals within 3 h. Animals in the bCPAP group consistently had lower PaCO_2_ values, and improved pH as early as 30 min after the bCPAP was started. This was also accompanied by improved oxygenation. This study suggested that bCPAP was capable of improving peripheral airway patency and decreased lung inhomogeneity, which was accompanied by a reduction in alveolar protein levels suggesting decrease in injury (36).

The degree of respiratory support afforded by a bCPAP system depends on its design. Diblasi et al. modified a traditional bCPAP system to increase the amplitude of the oscillations from ± 1 cm H2O to ± 2 cm H2O by changing the angulation of the bubbler. This change shifted the frequency band range from 9 to 20 Hz when the bubbler is angled straight down to 2–5 Hz when angled at 135°. When this change was used in juvenile rabbits, this increase in amplitude and drop in frequency range was associated with improved oxygenation, and a reduction in respiratory rates with stable PaCO_2_ (37).

The effect of CPAP surpasses that of just providing respiratory support, decreasing lung inflammation, and promoting lung growth. Edwards et al. demonstrated that invasive CPAP was associated with a reduction in periodic breathing epoch duration, and stabilized chemoreflex control in newborn lambs rendering it potentially useful in treating apnea of prematurity (38).

### Heated-Humidified High Flow Nasal Cannula (HFNC)

The ability of a HFNC system to provide respiratory support in neonatal models of lung disease was explored by Frizzola et al. Using term neonatal piglets injured with intravenous oleic acid, they demonstrated that CO_2_ clearance, oxygenation, and tracheal pressures increased in a flow dependent manner, with higher flows providing more respiratory support. Furthermore, the utilization of a single vs. double pronged approach to HFNC impacted the amount and type of respiratory support afforded at each flow rate; with 2 prongs improving oxygenation, and a single prong promoting more CO_2_ washout (39).

The aforementioned findings are also supported by a study conducted in healthy adult canines. The provision of HFNC at rates ranging between 0.4 and 2.5 L/kg/min, generated increasing oropharyngeal pressures in a flow-dependent manner. The range of pressures generated was wide with pressure differentials spanning 1.4 to 10.2 cm H2O during inspiration, and 4.1 to 12.9 cm H2O during expiration at flows of 2.5 L/kg/min. Furthermore, increasing flow was associated with an increase in oropharyngeal FiO_2_, PaO_2_, and end tidal O_2_. Interestingly, flows of 2 L/kg/min or less resulted in an increase in PaCO_2_, which may be related to the dual prong approach used (40).

The increase in PaCO_2_ when dual prongs are used with HFNC was again demonstrated in a retrospective study in canine patients presenting with hypoxemia. Animals that transitioned from traditional oxygen therapy to HFNC experienced an improvement in oxygenation with an increase in PaO_2_. This was accompanied by an increase in PaCO_2_ (41).

One of the possible explanations for the improved CO_2_ clearance with single-pronged HFNC at similar flow rates, is the increased leak through the second nostril (39), which could promote more laminar flow through the nasopharynx, allowing for improved CO_2_ washout.

Whether HFNC impacts inflammation in BPD models has yet to be elucidated.

### High Frequency Nasal Ventilation (HFNV)

Given the body of evidence supporting the use of continuous distending pressure to reduce lung injury and inflammation, while promoting lung development, newer modalities of support were sought. One such modality is HFNV which has yet to gain significant traction clinically. The earliest exploration of HFNV was by van der Hoeven et al. (42) which tested the modality in human neonates (42).

It wasn't until 2008 when Reyburn et al. utilized premature lamb models to study the biological impact of HFNV. Premature lambs delivered at 130 to 132 days were supported for 3 days on either invasive mechanical ventilation (IMV) or HFNV generated by a high-frequency flow-interrupter device from Percussionaire Inc. (Sand Point, ID). HFNV permitted normal alveolarization to continue as shown by improved radial alveolar counts, and secondary septal densities that were concordant with gestational development. A mechanism proposed by the team involves altered mesenchymal cell turnover, with HFNV promoting mesenchymal cell apoptosis and hence thinning of the distal airspace walls (43).

The importance of endodermal-mesodermal maturation in how HFNV supports lung development was studied by Rehan et al. in premature lamb models of BPD. Animals born prematurely were supported with either HFNV or IMV for 21 days. Animals in the HFNV groups had enhanced alveolar parathyroid hormone-related protein-peroxisome proliferator-activated receptor (PTHrP-PPARγ) signaling, a critical alveolarization pathway which has been implicated in alveolar-mesodermal maturation of the lung (44). The improvement in alveolarization observed in animals supported with HFNV was associated with reduced pressure and FiO_2_ requirements as demonstrated by Null et al. in lamb models of BPD (45).

Interestingly, non-sedated animals receiving non-invasive respiratory support often experience inspiratory laryngeal closure which limits the utility of NIMV (46). Contrary to NIMV, HFNV was found to not induce active laryngeal closure (47).

The need for a multidisciplinary approach to address lung injury and development was shown by Joss-Moore et al. in a premature lamb model of BPD. Despite the early use of HFNV, they demonstrated that nutritional restriction lead to alveolar simplification despite optimizing respiratory support (48).

These findings suggest that the use of HFNV within a multidisciplinary package to reduce BPD may be beneficial.

### Neurally Adjusted Ventilator Assist (NAVA)

The proprietary nature of NAVA and its restriction to Servo ventilators has limited its dissemination. The use of NAVA to provide non-invasive support in neonatal models of lung disease has not been explored. In a single study by Hadj-Ahmed et al. non-sedated term-born lambs without lung disease were supported with either NIMV (non-invasive pressure support) or NAVA using a nasal mask. The team was interested in exploring whether the enhanced synchrony affected upper airway dynamics. Indeed non-invasive NAVA support was not associated with active glottal closure contrary to NIMV (49).

In adult aspiration models of lung injury, non-invasive NAVA was shown to reduce lung injury scores, and allowed dynamic lung compliance to recover faster when compared to invasive provision of volume-control ventilation (50). Furthermore, when coupled with a low resistance leaky nasal interface, NAVA was capable of delivering pressure, unloaded the respiratory muscles, and maintained patient-ventilator synchrony (51).

Whether non-invasive NAVA confers any lung protection in animal models of BPD is unknown.

## Clinical Trials of Non-Invasive Support

### CPAP

CPAP is one of the most commonly used non-invasive support modalities in preterm infants. It can be used as an initial mode of support or following extubation.

#### CPAP as an Initial Mode of Support

The feasibility of studying delivery room application of CPAP (DR-CPAP) was explored by the National Institute of Child Health and Human Development Neonatal Research Network between July 2002 and January 2003 (52). Since then, multiple studies have examined the short-term and long-term effects of DR-CPAP, and this culminated in a 2016 Cochrane review concluding that the prophylactic use of DR-CPAP was associated with a significant reduction in need for mechanical ventilation, and a concomitant reduction in surfactant use. This report also noted a minor reduction in incidence of BPD at 36 weeks (53). These findings were corroborated by a study by Govindaswami et al. who demonstrated that consistent use of non-invasive supportive strategies including CPAP minimize the risk of intubation and increased the survival without major morbidities in very preterm infants (54).

In 2003 Narendran et al. were able to replicate the University of Columbia's experience in the application of bCPAP in the delivery room using nasal prongs rather than a face mask. Their early application of bCPAP was associated with a reduction in intubation in the delivery room, decreased the use of postnatal corticosteroid use, and a significant improvement in postnatal weight gain. Despite these findings they only experienced a statistically insignificant trend toward lower incidence of BPD (55).

An international randomized controlled trial to evaluate the efficacy of combining prophylactic surfactant and early CPAP in very preterm infants (CURPAP study) was designed to compare the administration of prophylactic vs. selective surfactant followed when nCPAP was used. The primary end point was the need for invasive ventilation in the first 5 days of life. After recruiting 208 premature 25 to 28 weeks' gestation infants, they concluded that prophylactic surfactant was not superior to nCPAP when combined with early selective surfactant in decreasing the need for ventilation and other morbidities of prematurity (56).

When Rojas et al. randomized 279 preterm infants with evidence of respiratory distress and requiring supplemental oxygen in the delivery room to intubation, very early surfactant, and extubation, followed by bCPAP or bCPAP alone. Infants receiving early surfactant and bCPAP without invasive ventilation were less likely to develop pneumothoraces nor require subsequent invasive ventilation (57).

In a Cochrane database systematic review on non-invasive respiratory support that included six older studies conducted between 1973 and 2007, the authors concluded that the application of continuous distending pressure (CDP) whether positive as in CPAP or negative as when applied through a chest cuirass, is associated with reduced respiratory failure and mortality, with an increased rate of air leaks (58). When more contemporary studies were evaluated through meta-analysis, Subramaniam et al. showed that the early application of CPAP is associated with a decrease in mortality and need for invasive ventilation (53).

#### Impact of CPAP on Development of BPD

In the CPAP or Intubation at Birth (COIN) trial, 610 extremely premature infants were randomized to either CPAP or intubation with mechanical ventilation at 5 min of life. The use of early CPAP was associated with a reduction of death or BPD at 28 days. However, this difference did not persist at 36 weeks corrected gestational age. Interestingly, the infants randomized to the CPAP group were only on CPAP for a median of 13 days (59). The larger Surfactant Positive Pressure and Oxygenation Randomized Trial (SUPPORT) that investigated the use of early intubation and surfactant therapy and compared it to early CPAP, did not demonstrate a significant reduction in the incidence of death or BPD at 36 weeks corrected gestational age. The use of CPAP however was found to reduce the need for intubation, postnatal corticosteroids, and length of invasive ventilation (60). In the Delivery Room Management Trial, the use of early CPAP also did not confer any statistical benefit toward reducing BPD when compared to early surfactant with rapid extubation, or to invasive mechanical ventilation (61).

However, when data from the aforementioned trials along with 4 more was combined in a metanalysis Cochrane Review by Subramaniam et al. the authors demonstrated the efficacy of CPAP in reducing BPD and the combined outcome of BPD or death when compared to mechanical ventilation (53).

#### Does the Bubbling of bCPAP Matter?

bCPAP is a relatively cheap and easy modality of respiratory support to deploy when compared to various nCPAP devices (55, 62). However, few studies directly compared the two.

Pelligra et al., compared ventilator-derived CPAP and bCPAP over 2 different time periods. They found that the implementation of bCPAP was associated with a significant reduction in the use of surfactant, postnatal steroids, and the duration of mechanical (63). In a randomized trial Tagare et al. demonstrated that bCPAP was more successful in managing early onset respiratory distress when compared to ventilator derived nCPAP (64). In a study comparing Jet CPAP to bCPAP, there was no difference in failure rates between the groups, suggesting that bCPAP is a viable alternative (65). When compared to CPAP delivered through the Infant Flow Device, bCPAP was associated with a shorter duration of non-invasive support (66).

Bubble CPAP appears to be safe, capable of reducing the need for mechanical ventilation, and reduces in-hospital mortality in low to middle income countries (67, 68).

### Non-invasive Mandatory Ventilation (NIMV)

Clinical studies exploring the utility of NIMV are difficult to interpret due to the different ventilators and interfaces used (Table 1 highlights some of the recent studies exploring NIMV that had recruited more than 100 patients, and the different devices and interfaces used).

**Table 1:** The Larger NIMV studies, their comparisons, and the different interfaces and ventilators utilized.

**Study**	**Comparison**	**Primary outcome**	**N**	**Synchrony**	**Interface**	**Device used for NIV**
Afjeh et al. (69)	NIMV vs. CMV	N/A	499	N/A	N/A	N/A
Baneshi et al. (70)	NIMV vs. CPAP	Survival	120	N/A	Nasopharyngeal prongs	Event medical ventilator
Bhandari et al. (71)	NIMV vs. CPAP	Death or BPD	469	Yes	N/A	Infant star
(Biniwale and Wertheimer (72)	NIMV vs. T-Piece	DR intubation	221	No	Neotech RAM cannula	N/A
Chen et al. (73)	NIMV vs. bCPAP	Initial intubation	129	No	N/A	Draeger babylog, stephan bubble CPAP
Dumpa et al. (74)	Synchrony	N/A	410	Yes	N/A	Infant star, bear cub 750 psv
Dumpa et al. (75)	CMV vs. NIMV vs. CPAP	Death or BPD	164	Variable	N/A	Infant Star, Bear Cub 750 psv
Esmaeilnia et al. (76)	NIMV vs. CPAP	Extubation failure	160	N/A	N/A	N/A
Kirpalani et al. (77)	NIMV vs. CPAP	Death or BPD	1009	Variable	N/A	N/A
Mehta et al. (78)	NIMV only	Initial intubation	240	No	Ackrad INCA prongs	Avea
Meneses et al. (79)	NIMV vs. bCPAP	Initial intubation	200	No	Short Bi-nasal prongs	Inter Neo vs. bCPAP
Millar et al. (80)	Ventilator NIMV vs. Bi-Level	Death or BPD	497	Both	N/A	Draeger Babylog, Bird VIP/VIP Gold, Evita 4/XL, Servo 300/900c/I, and Others
Oncel et al. (81)	NIMV vs. NCPAP	Initial intubation	200	No	Ackrad INCA prongs	SLE neonatal ventilator
Ramanathan et al. (82)	NIMV vs. NCPAP	Reintubation	108	No	Nasal or NP prongs	Avea, SiPAP
Shi et al. (83)	NIMV vs. NCPAP	Initial intubation	179	No	N/A	Drager babylog

#### NIMV as an Initial Mode of Support

NIMV has been shown to reduce the need for primary intubation when compared to CPAP (81–84). However, these findings were not consistently observed in others. In the studies by Li et al. and Meneses et al. the use of early NIMV did not decrease the need for mechanical ventilation when compared to CPAP (79, 85).

In the most recent Cochrane data base systematic review from 2016. Lemyre et al. consolidated the data from 10 trials exploring NIMV as an initial mode of support. The use of early NIMV was associated with a significantly reduced risk of meeting respiratory failure criteria and needing intubation when compared with early NCPAP in preterm infants with respiratory distress syndrome (86).

#### NIMV for Post-extubation Support

In the largest study exploring NIMV for respiratory support, Kirpalani et al. did not show a benefit to NIMV in preventing reintubation when compared to CPAP (77). When data from this study was pooled with 9 others conducted between 1999 and 2016, the use of NIMV was associated with a reduced incidence of extubation failure (87).

#### The Impact of NIMV on Developing BPD

In a relatively older study by Bhandari et al., comparing invasive ventilation to NIMV, the use of NIMV was not only feasible in supporting infants with RDS, but was also associated with a decrease in BPD (88). In a retrospective examination, Dumpa and colleagues. confirmed the advantage conferred by NIMV over invasive ventilation in reducing BPD rates, but did not demonstrate a similar advantage when NIMV was compared to CPAP (75). In a small single center randomized trial, Kugelman et al. compared infants receiving NIMV to others on nCPAP. Very low birth weight infants in the NIMV group had a 50% reduction in intubation and endotracheal ventilation when compared to nCPAP, and that resulted in a marked decrease in the incidence of BPD (89). A similar trend was seen in another small study by Badiee et al. which was exploring short term respiratory outcomes of NIMV when compared to CPAP. These trends however did not reach statistical significance (90). A larger international multi-centered study involving 1009 infants of <30 weeks of gestation did not find a difference in BPD rates between NIMV and CPAP, whether it was used as an initial mode of non-invasive support or a post extubation modality (77). A limitation of most of the aforementioned studies is not matching mean airway pressures (MAP) between the NIMV and CPAP groups. Buzzella et al. had previously demonstrated that higher CPAP pressures were associated with a reduced risk of CPAP failure (91). At this time, no studies have examined the long-term effects of NIMV with MAP-matched CPAP. However, when a SiPAP NIMV device was compared to MAP-matched CPAP in a cross-over study, the SiPAP device did not confer any benefits to CO_2_ removal or oxygenation (92).

A study by Millar et al. involving 455 extremely low birth weight infants, aiming to explore the impact of different devices used to deliver NIMV on the development of BPD, failed to demonstrate any differences in the incidence of BPD between dedicated flow drivers such as SiPAP, and ventilator derived support (80).

Interestingly, the timing of NIMV support does appear to impact the likelihood of developing BPD as shown by the two Cochrane reviews exploring the topic. When applied early, NIMV seems to confer a protective effect when compared to CPAP. This may be related to a demonstrated reduction in need for intubation. However, when NIMV is used post-extubation, this benefit no longer is observed, likely due to ventilator induced lung injury (86, 87).

#### Does NIMV Synchrony Matter?

The ability of a ventilator to synchronize during NIMV is dependent on its proprietary hardware and software, with some ventilators being incapable of synchronizing breaths at all, while others are faced by challenges related to leak compensation. Synchronization can typically be achieved through the use of flow-triggers or an abdominal capsule that measures abdominal excursion.

One of the earlier studies exploring synchrony of NIMV showed that it led to improved ventilator interactions and hence a reduction in work of breathing. Of note, the team had used the same ventilator for both asynchronous and synchronous NIMV (93). Subsequently a larger retrospective study was conducted by Dumpa et al. In their retrospective examination they concluded that synchrony did not affect short or long-term outcomes. However, the ventilators employed differed between the modes (74).

Interestingly, in a small study exploring the impact of synchrony on apnea of prematurity, Gizzi et al. demonstrated the efficacy that achieving synchrony has in reducing apneic episode burden (94).

When examining the effect of synchrony on delivered tidal volumes, Owen et al. concluded that in SiPAP-generated NIMV, tidal volumes were not impacted by synchrony. Furthermore, no tidal volume was delivered during apneic events at peak-pressure (95). These findings could be potentially explained by the limited ability of SiPAP drivers to generate a big difference in pressures between peak-pressure and PEEP.

A more structured approach to studying the impact of synchrony is needed while controlling for ventilator-dependent variables.

### Non-invasive Neurally Adjusted Ventilatory Assist (NIV-NAVA)

Given the improvements observed in peak pressures and FiO_2_ requirements when NAVA is utilized invasively, interest has peaked in its application for non-invasive support. Despite that, a limited number of studies have explored NIV-NAVA in preterm infants.

In an initial small cross-over study comparing NIV-NAVA and NIMV in 15 preterm neonates, NIV-NAVA was associated with more than a 3-fold improvement in patient-ventilator interactions (96). In a report by Stein and colleagues, they describe the use of NIV-NAVA to facilitate extubation of premature infants until they were transitioned to CPAP. Whether this benefit was due to the improved synchrony, proportional assist, or increased mean airway pressure is unknown (97).

Interestingly, in another retrospective study comparing NIV-NAVA to CPAP for post-extubation support, Lee and colleagues concluded that the use of NIV-NAVA was associated with a lower risk of reintubation within 72 h (98). However, when Yonehara retrospectively examined a cohort of preterm infants supported with NIV-NAVA or NIMV post-extubation, they did not show any differences in extubation failure between these two modalities (99).

None of the studies on NIV-NAVA reported any benefit regarding the development of BPD.

### Heated-Humidified High Flow Nasal Cannula (HFNC)

The use of heated and humidified high flow gas delivery through short nasal prongs has become popular among caregivers due to ease of administration, simple interface and less perceived discomfort to patients.

Initially, the study by Lavizzari et al. suggested that HFNC when used as an initial respiratory support modality in late preterm and term infants was not inferior to CPAP in regard to short-term respiratory outcomes (100). However, in the recently published HUNTER trial, among preterm babies of more than 31 weeks GA, the use of HFNC as an initial mode of support was shown to be inferior to CPAP and resulted in a significantly higher incidence of intubation (101). Furthermore, the HIPSTER multicentered randomized trial was cut short by request of the their independent data and safety committee after treatment failure was noted to occur twice as frequently in the HFNC group when compared to CPAP (102).

However, when used post-extubation, HFNC appeared to be non-inferior to CPAP despite a higher trend for treatment failure with fallback to CPAP (103). The utility of HFNC as a post-extubation modality was also demonstrated by Yoder et al. in a randomized trial. In their study, HFNC was not inferior to CPAP whether used as an initial support modality or post-extubation (104). However, their study recruited larger infants more than 28 weeks of gestation.

When exploring the impact HFNC has on BPD rates, the most recent Cochrane review had concluded that HFNC was associated with similar rates of BPD as CPAP. However, the review which was published in 2016, did not include either the larger HUNTER or HIPSTER trials, and had included studies that mostly focused on more mature preterms and term infants at a lower risk of developing BPD (105). Whether HFNC is comparable to CPAP or NIMV where extremely premature infants are involved is unknown.

### High Frequency Nasal Ventilation (HFNV)

Given that in animal models HFNV had favorable protective effects on lung development, and that it combined the benefits of distending pressure generated by CPAP with the increased CO_2_ clearance of high-frequency ventilation, HFNV needed to be explored as a feasible mode of support in human neonates.

The earliest clinical trial of HFNV dates to 1998. Twenty one premature infants who deteriorated on nCPAP were treated with HFNV to prevent intubation. This report demonstrated the capability of HFNV in reducing PaCO_2_ in most treated infants (42). This was followed a 3 patient case series in 2015 by Aktas et al. which highlighted the utility of HFNV in supporting premature infants post-extubation (106).

The short-term effects of placing infants on HFNV was explored in a multicentered cross over trial by Bottino et al. When compared to nCPAP, infants on HFNV developed lower transcutaneous PaCO_2_ values with a trend toward improved oxygenation (107).

In a single center retrospective study spanning a 5-year period by Maneenil et al., 199 neonates who were not initially intubated were supported with either HFNV or nCPAP. Infants in the HFNV group were of lower gestational age and had higher oxygenation indices indicating worse clinical status at recruitment. Despite these differences, HFNV was shown to be as effective as nCPAP in preventing reintubation (108).

In prospective randomized controlled trials by Zhu et al. and Iranpour and colleagues comparing HFNV to nCPAP, HFNV was shown to be superior to nCPAP in reducing the need for intubation and mechanical ventilation (109, 110). A reduction in grade 2 intraventricular hemorrhage was noted in one of the two trials (110).

In another study involving 124 infants born at younger gestational ages of 28–34 weeks, Malakian et al. demonstrated that supporting infants with HFNV lead to lower PaCO_2_ measurements, and a lower duration of non-invasive support when compared to nCPAP. There was no difference in need for intubation between the groups. Interestingly, none of the children involved in the study developed significant BPD (111).

To assess the utility of HFNV in managing infants post extubation, a randomized controlled trial by Chen et al. demonstrated that HFNV was superior to nCPAP in improving post extubation pCO_2_ measurements and reducing reintubation rates in infants ≤ 32 weeks of gestation. Furthermore, the length of stay was decreased by around 5.6 days. HFNV did not appear to influence other outcomes such as BPD, NEC, or Death (112).

When data from multiple trials was consolidated in a meta-analysis by Li et al., HFNV was associated with enhanced CO_2_ clearance and decreased the risk for intubation consistently when compared to CPAP. However the relatively smaller numbers of recruited infants in the included 8 studies and their larger gestational ages limited the ability to delineate whether HFNV affects long-term outcomes such as BPD (113). A large prospective multi-centered trial is currently underway and aims to examine short and long-term outcomes (114).

## Non-Invasive Support Failure

The diagnosis of non-invasive support failure relies on various subjective and objective factors with some of the cutoffs used being arbitrary in nature. It is therefore imperative to consider the definitions used to determine failure. The importance of standardizing definitions of CPAP failure and respiratory management is demonstrated by a published quality improvement initiative by Birenbaum et al. Using clear definitions of CPAP failure, and multiple QI cycles, their team was able to increase the utilization of CPAP on admission from 13.7 to 64.4%, reduced mechanical ventilation in the first 72 h from 71.2 to 35.6%, and thereafter decreased their incidence of BPD from 46.5% in 2002 to 20.5% in 2005 (115).

One of the important factors impacting the establishment of functional residual capacity and improving dynamic respiratory compliance, and hence affecting oxygen requirements is the pulmonary surfactant pool. In preterm lambs, Mulrooney et al. found that surfactant pool size negatively correlated with the risk of CPAP failure, and that only small amounts of endogenous surfactant are needed for CPAP success (116). These findings were recently corroborated in humans by a study conducted by Raschetti et al. In prospectively followed infants, gastric aspirates were assessed for lamellar bodies; a correlate of the endogenous surfactant pool. They confirmed the positive effect prenatal corticosteroids have on enlarging the endogenous surfactant pool, and that a larger pool was moderately associated with CPAP success (117).

Given that lung development and surfactant production correlate well with the stage of lung development, infants born at a later age of gestation with larger birth weights are less likely to “fail” CPAP challenges (118, 119). However, we would like to caution against the use of gestational age or birth weights as reasons not to pursue a non-invasive support strategy as an initial management.

In a cohort of 11,684 infants initially managed with CPAP, the development of pneumothorax was associated with CPAP failure. Furthermore, CPAP failure was associated with increased odds of death or development of BPD (119).

The form of non-invasive support administered can influence “failure.” The provision of NIMV has been shown to be superior to CPAP in a number of clinical studies (120–122).

Another rare reason for non-invasive respiratory support is nasal trauma which precludes ongoing application of some nasal interfaces. Caring of infants on non-invasive support requires meticulous nasal care to prevent nasal septal injury (123).

## Nasal Injuries

Nasal pressure injuries due to interface placement continue to be a major issue with non-invasive respiratory support. They are seen most commonly with nasal prongs, and the rates at which they occur will depend on the type of prongs used, along with the method of securing those prongs to the head (124). Infants under 30 weeks of gestation are at higher risk of nasal injury when compared to term and near term infants (125). Nasal injuries occur due to excessive pressure exerted by the prongs on surrounding tissue, and interfaces that do not form a seal such as HFNC are less likely to cause injury (126). The use of nasal masks rather than prongs should be considered as a means to reduce nasal breakdown. In a recent meta-analysis of available studies King et al. showed that nasal masks carried a lower risk of failure within 72 h, and were associated with less injury when compared to bi-nasal prongs (127).

Given the ongoing potential for pressure injury, meticulous nursing care and routine application of hydrocolloid barrier dressing should be performed. In a randomized controlled trial, the use of such barriers has been shown to reduce the likelihood of any nasal injury by 39.7% (128).

## Other Outcomes

The clinical studies of NIV have addressed mostly short-term outcomes like need for mechanical ventilation, development of BPD and mortality. Neuro developmental Impairment (NDI) at 1 and 2 years of age have not been well-studied in a prospective manner. In a retrospective study from the Netherlands the shift to restricted use of mechanical ventilation and reliance on CPAP for respiratory support was associated with a lower risk for NDI and the composite outcome of NDI or death (129). The SUPPORT trial was the only large trial which has reported prospectively the 18–22 month neurodevelopmental outcomes, and found no significant differences in the composite outcome of death or neurodevelopmental impairment at 18–22 month corrected age among extremely premature infants randomly assigned to early CPAP or early surfactant administration and to a lower or higher target range of oxygen saturation (130). There may be some challenges in designing studies with NDO as primary outcome due to various confounding variables.

## Discussion

Prevention of lung injury starts with respiratory management in the delivery room. The choice of how to deliver distending pressure and positive pressure ventilation early on can have profound long-term effects. This was highlighted in premature lambs when six large breaths given early on were associated with worse pulmonary mechanics (131). The pioneering work of Wung et al. at Columbia University in New York highlights the importance of a structured approach to initial resuscitation with early CPAP and strict intubation guidelines in reducing lung injury and BPD (10, 132, 133). This has been reproduced successfully in multiple neonatal intensive care units in North America (115, 134). The feasibility of implementing early CPAP in the delivery room has been verified by a number of studies (52, 135, 136). This was not limited to affluent institutions and locales, with multiple studies demonstrating the efficacy of utilizing early CPAP in resource limited settings (137, 138).

Since occasionally infants may require intubation for early resuscitation and surfactant administration, gentle ventilation strategies and early extubation are paramount in reducing lung injury. In a retrospective study by Friedman et al. a combined approach of bCPAP, early surfactant therapy, and rapid extubation was associated with a 27% reduction in BPD (139). Furthermore, delaying extubation to days 4 to 7 of life increases the risk of developing BPD by 70% in infants born at ≤ 28 weeks (140). The impact of early extubation can also result in shorter lengths of stay (141).

Given that surfactant administration is one of the most common indications for intubation, exploring non-invasive means of its delivery had sparked the interest of practicing neonatologists. In animal studies of nebulized surfactant, lung deposition of surfactant varied widely, was a fraction of what was nebulized, and favored dependent lung segments (142). Moreover, most clinical studies investigating nebulization of surfactant utilized jet nebulizers and did not demonstrate any benefits (143–145). However, a recent randomized trial utilizing a custom vibrating membrane nebulizer and Poractant alfa (Chiesi Farmaceutici SpA, Parma, Italy) demonstrated the feasibility of nebulized surfactant when used in conjunction with bCPAP in reducing intubation within 72 h when compared to bCPAP alone (146). These findings will however need to be confirmed in a larger trial, and its effect on BPD needs to be elucidated.

Given the delineated biologic benefits of early distending pressure, regardless of form, the choice of post-extubation support modality should focus on its ability to provide said distending pressures reliably. The low cost of bCPAP compared to other devices renders it an attractive starting point for many providers and units including those in resource limited settings (68, 147).

The use of NIMV as a primary support modality post-extubation, or as rescue after CPAP failure in an attempt to prevent reintubation appears to be a feasible approach as shown by a number of clinical studies (78, 79, 81, 89, 148, 149). Studies exploring how NIMV affects the development of BPD when compared to CPAP have shown conflicting results; these disparities are likely related to study design, timing of intervention, and inherent differences in interfaces and devices. How HFNV compares to NIMV is yet to be evaluated, but it appears to be superior to nCPAP in supporting premature infants with improved CO_2_ clearance and reducing the need for intubation (106–109).

The implementation of HFNV in NICU's in the United States and a significant portion of Western Europe has been limited due to the lack of clinically approved devices such as the medinCNO (medin Medical Innovations, GmbH, Germany) in the region. Devices such as high-frequency jet ventilators could be adapted to provide HFNV, whereas circuit limitations of the 3100A and 3100B oscillators (Vyaire Medical INC, formerly Carefusion) render such adaptations difficult.

Given the paucity of data surrounding the use of HFNC in animal models of BPD, the haphazard degree of distending pressure generated by these devices, and recent concerns raised regarding its use as a primary support modality or as a step down therapy, we would like to caution against the use of HFNC as a replacement of proper distending pressure (150–152). Furthermore, cost analysis of patients enrolled in the HIPSTER trial comparing CPAP to HFNC was heavily in favor of using any form of CPAP over HFNC (153). HFNC may have utility in supporting patients with established lung disease, in whom the goal of therapy no longer is prevention of BPD.

The question of how long distending pressure needs to be applied to promote lung development has somewhat been elusive in clinical trials, with most opting to wean off to a nasal cannula at arbitrary time-points. Animal data suggests that prolonged support with distending pressure whether it be bCPAP or HFNV for at least 2 to 4 weeks may be needed to preserve lung development (9, 34, 44, 45).

Maintaining interfaces in a proper position while ensuring the integrity of nasal skin requires ample education, proper staffing, and meticulous nursing care. Furthermore, the success of a non-invasive support program requires buy-in from all staff, along with adherence to standardized practices. Standardized practices would include strict intubation criteria, extensive use of CPAP as a first line modality in the delivery room and NICU, the use of NIMV as a second-line supportive modality, and relegating intubation and mechanical ventilation to a last-resort. The teams would then work to extubate these infants as soon as possible to limit ventilator induced lung injury, and likely use post-extubation NIMV to reduce extubation-failure, followed by a transition to CPAP. The application of continuous distending pressure should continue for as long as possible until the child can be weaned successfully to room air. The use of HFNC or regular nasal cannula should be limited to premature infants with established lung disease.

We the authors would like to posit a framework for designing and examining future studies of non-invasive respiratory support, where one will take into consideration the interfaces used, the devices used to deliver pressure and whether they're capable of leak compensation or synchrony, and duration of therapy. Without examining and controlling these factors, it is difficult to draw definitive conclusions surrounding any form of non-invasive respiratory support. Examination of HFNV, bubble NIMV devices, HFNC, MAP-matched CPAP and NIMV, and NIV-NAVA under the aforementioned framework are need.

## Author Contributions

IS and SK contributed equally to the writing of the first draft, and its revisions.

## Conflict of Interest

The authors declare that the research was conducted in the absence of any commercial or financial relationships that could be construed as a potential conflict of interest.
